# Thymoquinone protects against statin-induced cerebellar damage, apoptosis, and hepatic cytotoxicity in rats

**DOI:** 10.1038/s41598-026-50278-8

**Published:** 2026-05-26

**Authors:** Salwa M. Ouies, Yahia A. Amin, Noha A. Ragab, Zahraa M. Ismae, Mohamed A. Hassany, Basma T. Abd elhameed, Rehab G. M. Hassan, Walaa I. Mohammed, Maha Abd-El Baki Ahmed

**Affiliations:** 1 Department of Human Anatomy and Embryology, Faculty of Medicine, Sohag University, Sohag, Egypt; 2 Department of Theriogenology, Faculty of Veterinary Medicine, Aswan University, Aswan, 81528 Egypt; 3 Department of Internal Medicine, Faculty of Medicine, Aswan University, Aswan, Egypt; 4 Department of Pathology, Faculty of Medicine, South valley University, Qena, Egypt; 5 Department of Public Health, Faculty of Medicine, South Valley University, Qena, Egypt; 6 Department of Clinical Pharmacology, Faculty of Medicine, Sohag University, Sohag, Egypt; 7 Department of Human Anatomy and Embryology, Faculty of Medicine, South Valley University, Qena, Egypt

**Keywords:** Simvastatin, Liver, Cerebellum, Thymoquinone, Biochemistry, Cell biology, Diseases, Drug discovery, Medical research, Neuroscience

## Abstract

Statins are a class of drugs commonly prescribed to lower cholesterol and low-density lipoprotein (LDL) cholesterol levels, as well as to reduce morbidity and mortality associated with cardiovascular illnesses. However, statins are associated with potential side effects, such as muscle pain, liver problems, and neurological or psychiatric disturbances. Therefore, a protective medication is required to mitigate the harmful effects of statins. Thymoquinone (THQ), found in *Nigella sativa*, has several medicinal benefits and protective effects. The current study aimed to investigate the effects of simvastatin on cerebellar and hepatic tissues, focusing on cellular apoptosis, using light and electron microscopy, morphometric analysis, and immunohistochemical techniques. Additionally, the study evaluates whether thymoquinone can protect against simvastatin-induced histological and ultrastructural damage in hepatic and cerebellar tissues. Thirty adult male Wistar albino rats were divided into three equal groups. Group I was the control group (Ctrl group). Group II (statin group) received simvastatin (20 mg/kg/day) orally for 8 weeks. Group III (statin + THQ group) received concomitant treatment with simvastatin (20 mg/kg/day) and thymoquinone (10 mg/kg/day) for 8 weeks. Tissue specimens were taken from the liver and cerebellum of all animal groups and processed for light and electron microscopic examination. An immunohistochemical investigation for caspase-3 was performed. The molecular and granular layer thickness, number of Purkinje cells, central vein diameter, hepatocyte nuclear diameter, and optical density of anti-caspase-3-positive cells were measured in both the cerebellum and the liver in all experimental groups. Results showed that cerebellum from simvastatin-treated animals showed perinuclear halos in cells within the molecular layer. Purkinje cells appeared disorganized, irregularly arranged, shrunken, with irregular outlines, hyperchromatic nuclei, and vacuolated cytoplasm. Moreover, the granular cell layer appeared shrunken with irregular hyperchromatic nuclei. In addition, decreased thickness of both the molecular and granular layers, as well as a reduction in the number and diameter of Purkinje cells, was observed and confirmed by morphometric analysis. Liver tissue showed shrunken hepatocytes, widened blood sinusoids, widened central veins, and inflammatory cell infiltration. Furthermore, strong positive expression of caspase-3 was observed in both the cerebellum and liver. However, all these histological, ultrastructural, and morphometric changes were markedly decreased after the addition of thymoquinone. It can be concluded that simvastatin induces significant degeneration in both the cerebellar cortex and liver tissue, primarily due to increased oxidative stress and reduced antioxidant protection. Thymoquinone exerts protective effects against this oxidative damage in both organs and alleviates degenerative changes, suggesting that it may be recommended for hypercholesterolemic patients on statins.

## Introduction

Typically, statins are used to prevent and treat coronary heart disease, as they are the most effective medications for lowering LDL cholesterol and are also used to treat hyperlipidemia in people with chronic liver disorders. However, statins can cause side effects, including mitochondrial damage, increased production of reactive oxygen species (ROS), and the formation of reactive metabolites^1^.

Moreover, hepatotoxicity and myopathy are the most frequent side effects associated with statins. Animal studies have shown that liver toxicity and necrosis in hepatic cells are observed with higher statin dosages^2^. Mitochondrial dysfunction is a primary explanation for the mechanism of statin-induced hepatotoxicity. Mitochondrial toxicity assays show a significant increase in mitochondrial superoxide following statin treatment. Consequently, this increase leads to significant mitochondrial impairment. Additionally, statins’ ability to stimulate apoptotic cell death is another important factor contributing to statin-induced hepatotoxicity^3^.

Statins have been reported to cause neurological side effects, such as dementia, cognitive decline, and Alzheimer’s disease^4^. In addition, increased numbers of plaques in the hippocampus and cerebral cortex have been detected^5^. Moreover, simvastatin abrogated the expression of pro-inflammatory molecules, blocked glial cell activation, and was associated with experimentally induced Parkinson’s disease^6^.

Several natural plants with protective properties are now used in the treatment of diseases and to mitigate the side effects of therapeutic drugs and various toxicities^7–10^. One of these natural plants is Nigella sativa. The most prevalent component in *Nigella sativa* seed essential oil is thymoquinone (THQ), which possesses many of the plant’s characteristic properties. It has anticancer, estrogen receptor (ER)-lowering, anti-inflammatory, and antioxidant effects^11,12^. Moreover, it has neuroprotective, hepatoprotective, cardioprotective, and protective roles against testicular damage^13,14^.

Thymoquinone was found to reduce neuroinflammation and neurodegeneration^15^ by decreasing oxidative stress and increasing antioxidant capacity, thus providing neuroprotective efficacy^16^. Previous research evaluating the efficacy of THQ in treating epilepsy showed that THQ protects against damage to the hippocampus and cerebral cortex^17^.

Thymoquinone is characterized by high pharmacological activity against inflammation, toxins, and oxidative damage. Additionally, it may play a significant neuropharmacological role in the treatment of neurological conditions^18^. Numerous illnesses, including Parkinson’s disease, epilepsy, depression, and Alzheimer’s disease, have been linked to the possible neuropharmacological protective effects of THQ^19^. The combined protective effects of thymoquinone on both hepatic and cerebellar histology and ultrastructure have not been demonstrated in any previous study. Likewise, the characteristics of hepatic cells and cerebellar layers, as well as the cellular parameters of both organs, have not been simultaneously evaluated.

The current study aimed to investigate the effects of simvastatin on cerebellar and hepatic tissues, focusing on cellular apoptosis, using light and electron microscopy, morphometric analysis, and immunohistochemical techniques. Additionally, the study investigated whether thymoquinone can protect against simvastatin-induced histological and ultrastructural damage in hepatic and cerebellar tissues.

## Materials and methods

### Drugs and ethical approval

Statins were represented by simvastatin (Zocor tablets, 20 mg), which was obtained from Global Napi Pharmaceuticals, Egypt. Thymoquinone was represented by an orange crystalline powder obtained from Sigma-Aldrich. The study protocol was approved by the Institutional Research Committee at the Faculty of Medicine, South Valley University, Qena, Egypt, with ethical approval code: SVU-MED-ANA001-1-24-3-830, thereby ensuring compliance with recognized ethical standards. All animal procedures complied with the ARRIVE guidelines and were carried out in accordance with the U.K. Animals (Scientific Procedures) Act, 1986, and associated guidelines, as well as EU Directive 2010/63/EU for animal experiments.

### Animals

**Fig. 1:**
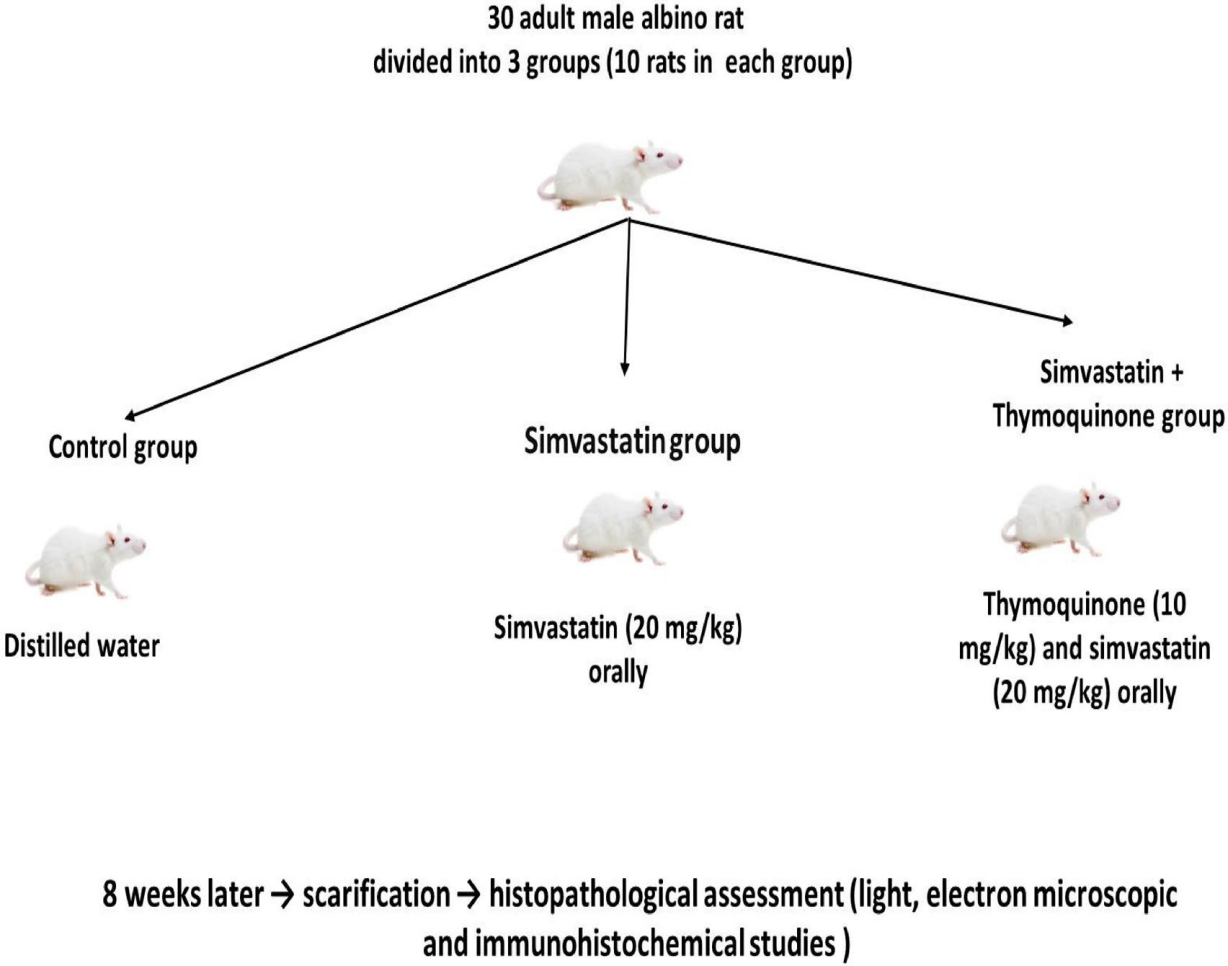
Summarized study design.

Thirty adult male Wistar albino rats (180–190 g), aged 3 months, were used in this study. Animals were acquired from the animal facilities at the Faculty of Medicine, South Valley University. Rats were maintained at ambient temperature with 55–60% humidity and a standard 12-hour light/12-hour dark cycle. They were kept under sanitary conditions, housed in metal cages with wood shavings as bedding, provided with ad libitum access to food, and allowed unrestricted access to water. Rats were randomly divided into three equal groups (10 rats per group). Group I was the control group (Ctrl), which received distilled water orally via an intragastric tube for 8 weeks. Group II was the Statin group, which received simvastatin (20 mg/kg/day) dissolved in distilled water and administered orally once daily via an intragastric tube for 8 weeks^20^. Group III was the statin + THQ group, which received both simvastatin (20 mg/kg/day) and thymoquinone (10 mg/kg/day, after dissolution in corn oil) via an intragastric tube for 8 weeks^21^. The summarized study design is shown in Fig. 1.

### Sample collection procedures

At the end of the experiment, the rats were anesthetized using inhalation of 3% isoflurane anesthesia^22^; subsequently, relevant tissues and organs were rapidly harvested. The cerebellum and liver from each rat were removed and processed for light microscopic (LM) and transmission electron microscopic (TEM) examination^23^.

### Light microscopic examination

The specimens for light microscopic investigation, after being fixed in 10% buffered formalin, were processed into 5 μm paraffin sections and stained with hematoxylin and eosin (H&E) to examine the general histological structure^24^.

### Immunohistochemical investigation

After removal of paraffin from 5 μm sections, rehydration and washing with phosphate-buffered saline were performed. Sections were soaked overnight with an anti-caspase-3 antibody (an apoptosis marker); a rabbit polyclonal anti-caspase-3 antibody (dilution 1:200; PA1-26426, Abcam, USA) was used as the primary antibody. A positive reaction was defined as brown cytoplasmic coloration. As a positive control, a section of a human tonsil was utilized^25^. The same technique was employed to obtain negative controls, except that the primary antibodies were omitted. Finally, Mayer’s hematoxylin was used to counterstain all immunostained slides.

### Ultrastructure preparations

Firstly, small sections of the cerebellum and liver (1 × 1 mm thick) were fixed in 5% glutaraldehyde and then post-fixed in 1% osmium tetroxide. Subsequently, semithin sections of 1 μm were prepared, stained with 1% toluidine blue in borax, and examined under a light microscope. Secondly, ultrathin sections of 50 nm were prepared using an ultramicrotome, mounted on copper grids, and stained with lead citrate and uranyl acetate^26^. The examination was conducted at the Electron Microscope Research Laboratory Unit of South Valley University. The sections were examined using a JEOL JEM-100CX II transmission electron microscope and then photographed.

### Morphometric analysis

Five slides were chosen from each animal. For each measured parameter, five non-overlapping fields were selected from each slide. Fields were selected by targeting representative, high-quality, and relevant areas of the tissue section. The group mean was estimated by averaging the individual means.

The investigated parameters were as follows:

- Mean thickness of the molecular layer of the cerebellar cortex in H&E-stained sections (×200).
- Mean thickness of the granular layer of the cerebellar cortex in H&E-stained sections (×200).
- Mean number of Purkinje cells in H&E-stained sections (×400).
- Central vein diameter (×200) and hepatocyte nuclear diameter (TEM ×25,000).
- Mean optical density of anti-caspase-3-positive cells in the cerebellum and liver (caspase-3 immunostained sections ×400).

### Statistical analysis

The values were expressed as mean ± SD. A one-way ANOVA followed by Tukey’s post hoc test was used to determine statistical differences between groups, the analysis was blinded. A P-value of less than 0.05 was considered statistically significant.

## Results

No signs of morbidity or mortality were observed in any of the three groups during the study period.

### Light microscopic examination of the cerebellum (H&E staining)

**Fig. 2:**
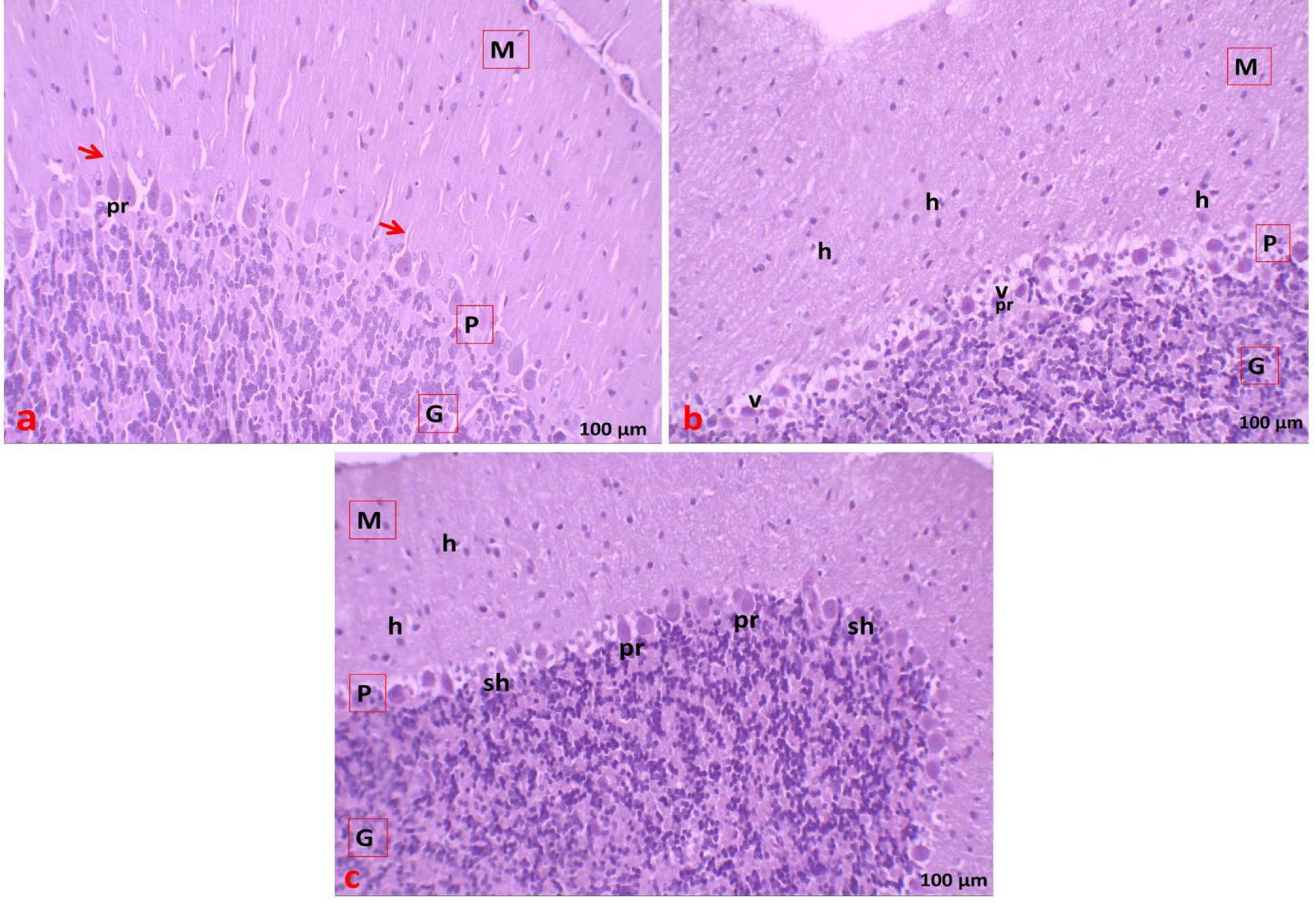
**(a)** Section of Cerebellar cortex of control group showing the three layers in the order: outer molecular (M), middle Purkinje (P), inner granular (G). Nerve fibers (red arrow) extended from the Purkinje cells passing through the molecular layer. **(b)** Section of Cerebellar cortex of statin group showing: outer molecular (M) with perinuclear haloes around cells (h), middle Purkinje cell layer (P) showing shrunken dark stained cells (pr) surrounded by multiple vacuoles(v), inner granular (G) layer with dark stained granule cells. **(c)** Section of Cerebellar cortex of Statin + THQ group showing molecular (M), with perinuclear haloes around cells (h). Purkinje layer (P) showing normal appearance of most cells (pr), with shrunken in some cells (sh). Granule cell layer (G) showed normal appearance (H&E, ×200).

**Fig. 3:**
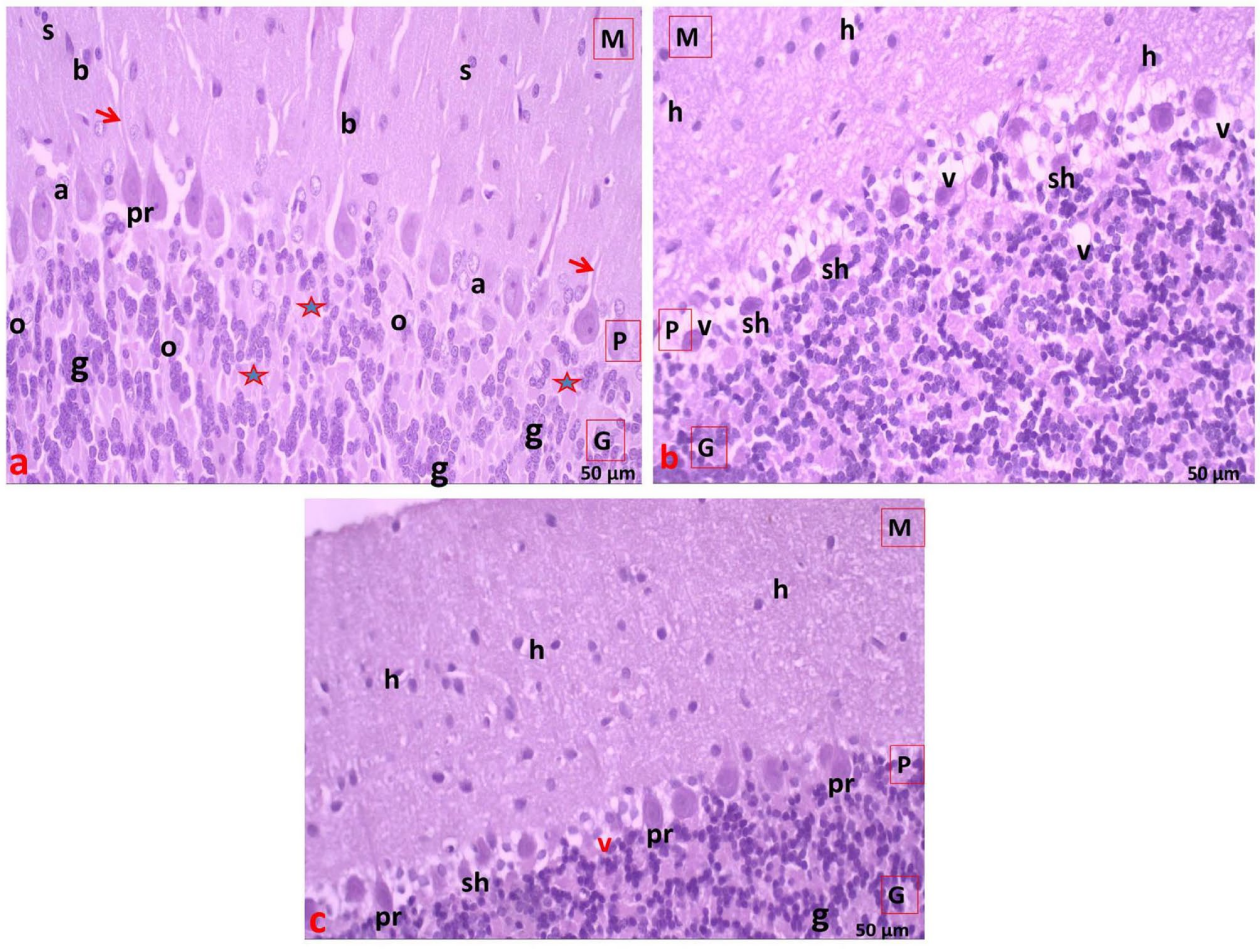
**(a)** Section of Cerebellar cortex of control group showing molecular layer (M) with small cells toward outside; stellate (s) and basket (**b**) cells; scattered cells near Purkinje cells. Purkinje cell layer (P) appeared with large “flask-shaped” cells (pr) oriented in one row, with nerve fibers (red arrow) extending outward, in between appear pale Bergmann astrocytes (a). Granule cell layer (G) formed of aggregations of deeply stained granule cells (g), large vesicular Golgi type II (o) with empty areas in-between (stars). **(b)** Section of Cerebellar cortex of Statin group showing: molecular layer (M) with perinuclear haloes around stellate cells and basket (h) cells. Purkinje cell layer (P) showing darky stained shrunken cells (sh) with multiple vacuoles (v). Granule cell layer (G) showed vacuoles (v). **(c)** Section of Cerebellar cortex of Statin + THQ group showing molecular layer (M) with normal cells in most areas and perinuclear haloes around (h) some cells. Purkinje cell layer (P) showing normal cells (pr), with some cells (sh) appear abnormal with vacuoles in-between cells. Granule cell layer (G) showing normal appearance of granule cells (g) which appear aggregated into groups. (H&E, ×400).

In the control group, H&E-stained cerebellar sections showed the three layers of the cerebellar cortex arranged regularly; the outer molecular layer, the middle Purkinje cell layer, and the inner granular layer (Fig. 2a). Two types of neuronal cells were observed in the molecular layer: stellate cells (smaller cells toward the outer surface) and basket cells (scattered cells near Purkinje cells). The Purkinje cell layer consisted of large, flask-shaped cells aligned in a single row, encircled by pale Bergmann astrocytes. The granular layer appeared as tightly packed granule cells with deeply stained nuclei, with few large vesicular Golgi type II cells interspersed (Fig. 3a).

In the statin-treated group, the cerebellar cortex exhibited vacuolated areas involving all cortical layers. Most cells of the molecular layer displayed perinuclear halos. The Purkinje cell layer showed disrupted, shrunken Purkinje cells with dark cytoplasm and pyknotic nuclei, surrounded by vacuolated areas. The granular layer contained darkly stained granule cells with cellular aggregates and interspersed vacuoles, while Golgi type II cells exhibited vacuolated cytoplasm (Figs. 2b and 3b).

In the statin + THQ group, most cells in the molecular layer appeared normal, with only a few showing perinuclear halos. Purkinje cells were largely normal, although some cells appeared shrunken with vacuolated areas in specific spaces. The granular layer appeared normal in most regions (Figs. 2c and 3c).

### Light microscopic examination of the liver (H&E staining)

**Fig. 4:**
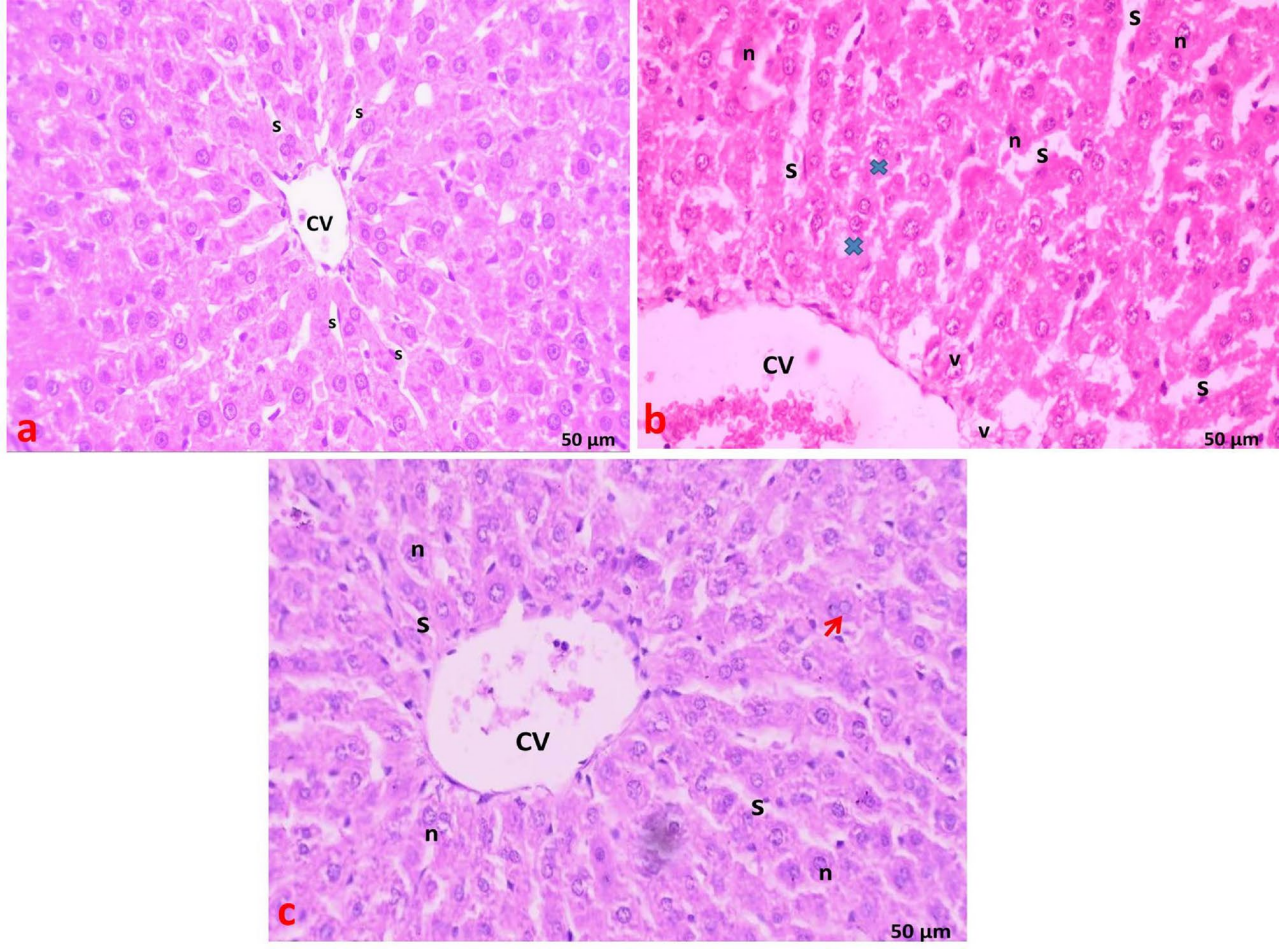
**(a)** Section of liver of control group showing hepatic lobule with cords of normal hepatocytes radiating from the central vein (CV). Narrow blood sinusoids (S) are radiating in between liver cords. **(b)** Section of liver of Statin group showing marked dilatation of the central vein (CV), loss of normal liver architecture (star), dilated blood sinusoids (S). Hepatocytes appear with ill-defined boundaries (**×**), condensed nuclei (n) and vacuolated cytoplasm. **(c)** Section of liver of Statin + THQ group showing normal central vein (CV) with hepatocytes radiating from it, most hepatocytes have acidophilic cytoplasm and rounded vesicular nuclei(n), binucleated cells( arrow) and normal narrow blood sinusoids (S) also seen. H&E; X 400.

**Fig. 5:**
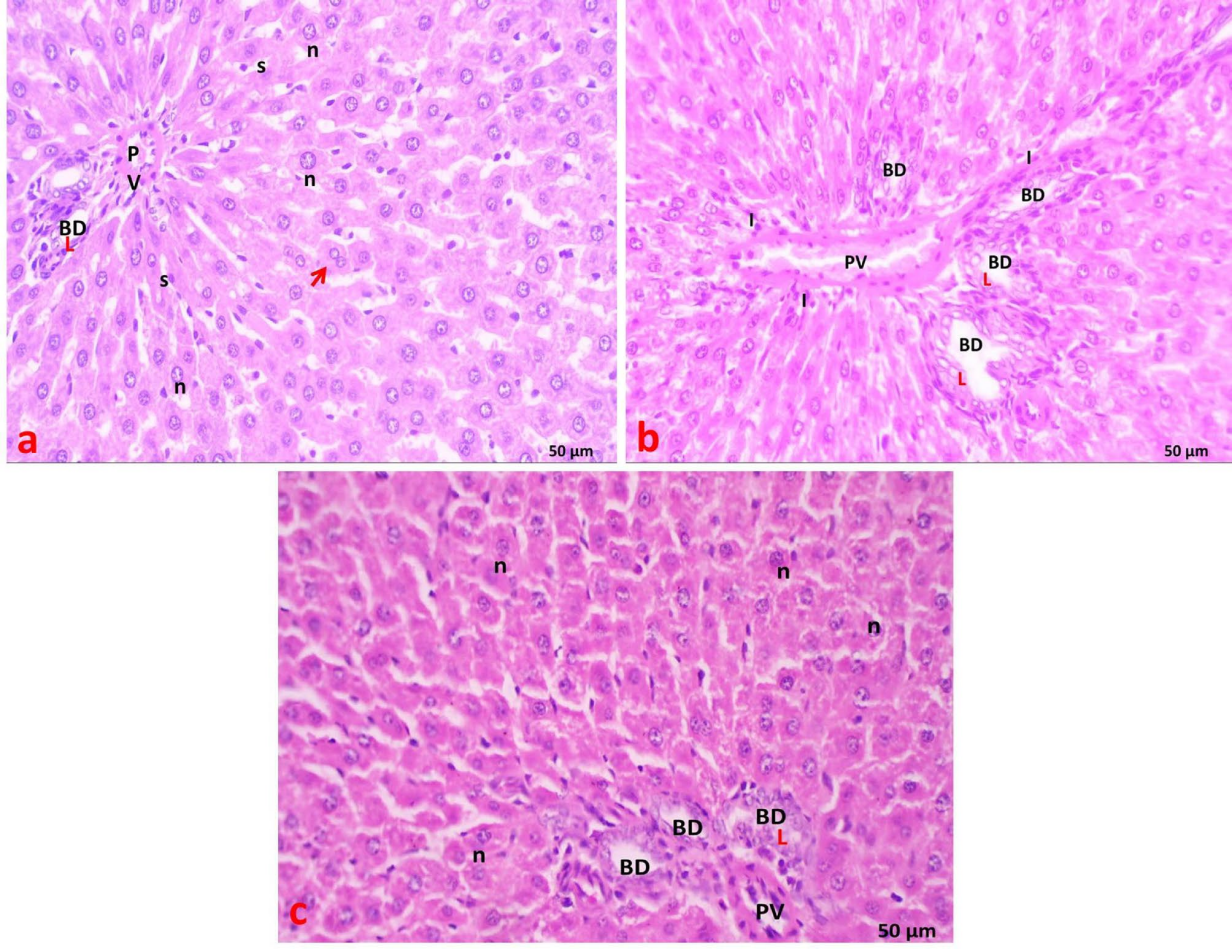
**(a)** Section of liver of control group showing the portal area with the portal vein (PV) and a bile duct (BD) lined with single cuboidal cells. Hepatocytes appear with rounded vesicular nuclei (n), binucleated cells are also visible with Narrow blood sinusoids (S) are radiating in between liver cords. **(b)** Section of liver of Statin group showing dilated portal vein (PV), many bile ducts (BD) with thick Lining (L), and few inflammatory cell infiltrations (I) in the portal area with marked loss of normal liver architecture (star). **(c)** Section of liver of Statin + THQ group showing portal vein (PV), most hepatocytes have acidophilic cytoplasm and rounded vesicular nuclei(n), still bile duct (BD) proliferation with thick Lining(L) appear H&E X 400.

In the control group, each hepatic lobule consisted of a central vein surrounded by densely packed cords of hepatocytes arranged radially. Each hepatic cord comprised polygonal hepatocytes with rounded vesicular nuclei and acidophilic cytoplasm. Additionally, binucleated cells were observed, with endothelial cell-lined radiating blood sinusoids between the hepatic cords (Fig. 4a). The portal area contained a bile duct lined by solitary cuboidal cells with dark, rounded nuclei and a portal vein with a large lumen and thin wall (Fig. 5a).

In the statin group, there was marked loss of normal liver architecture with variable hepatocellular changes. Some hepatocytes displayed poorly defined boundaries, condensed (dark-stained) nuclei, and vacuolated cytoplasm within the hepatic lobules. Widening of blood sinusoids and dilated central veins were noted (Fig. 4b). In the portal area, dilated portal veins, prominent cellular infiltration, thickened bile duct walls, and bile duct proliferation were observed (Fig. 5b).

In the statin + THQ group, varying degrees of improvement were noted compared to the statin group. Many hepatocytes resembled those of the control group, with acidophilic cytoplasm, rounded vesicular nuclei, and some binucleated cells. The central vein and blood sinusoids appeared normal. However, bile duct proliferation persisted (Figs. 4c and 5c).

### Immunohistochemical examination of the cerebellum and liver

**Fig. 6:**
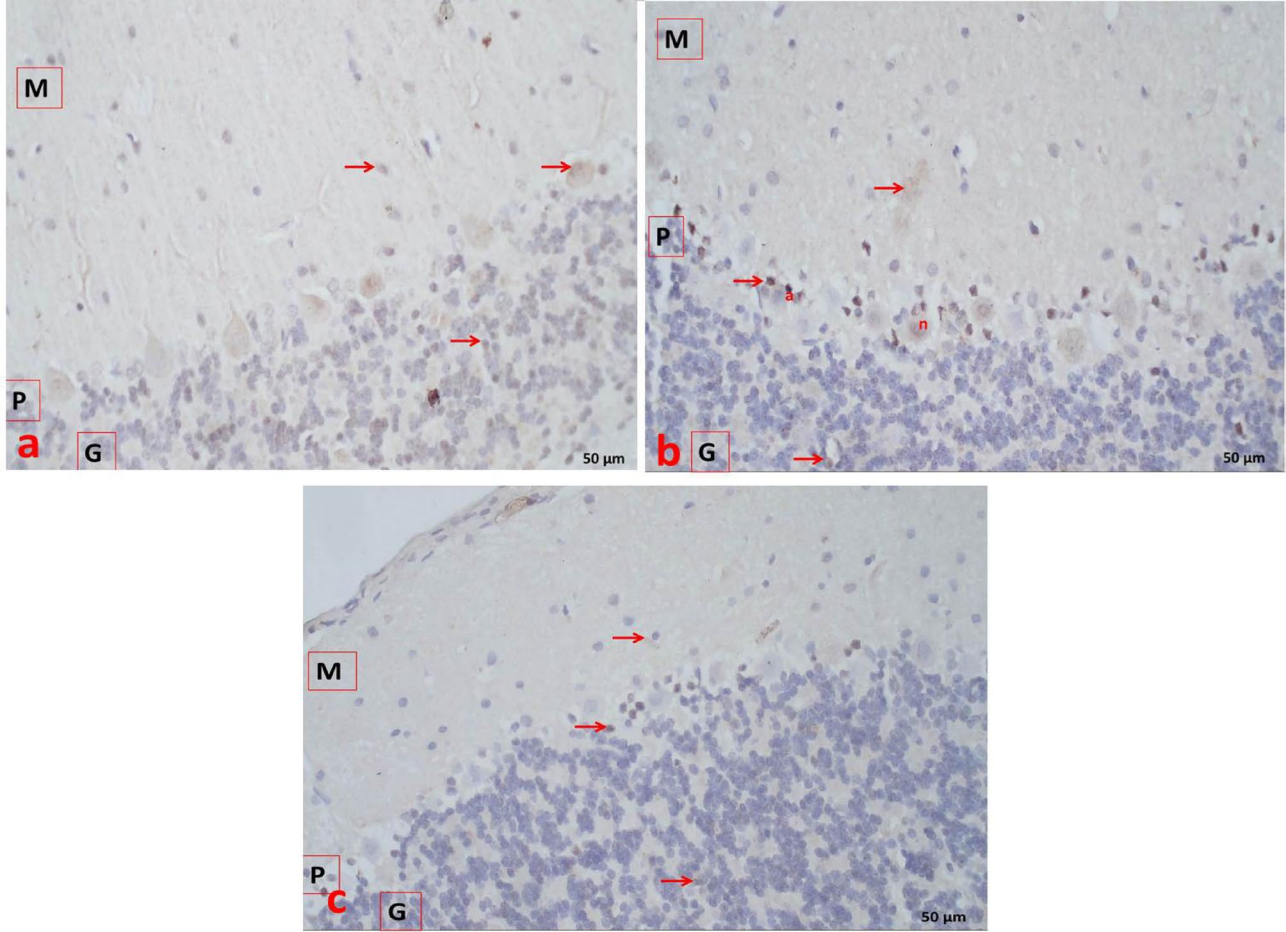
**(a)** Section of Cerebellar cortex of control group showing faint cytoplasmic immunoreaction (arrows) for caspase-3 in molecular layer (M), purkinje cells in the purkinje cell layer (P) & moderate in granule cell layer (G). **(b)** Section of Cerebellar cortex of Statin group showing faint positive cytoplasmic (arrows) and nuclear (n)immunoreaction for caspase-3 in molecular layer (M), strong in Bergmann astrocytes (a) and Purkinje cells in the Purkinje cell layer (P) & faint in Granule cell layer (G). **(c)** Section of Cerebellar cortex of Statin + THQ group showing very faint positive cytoplasmic immunoreaction (arrows) for caspase-3 in molecular layer (M), Bergmann astrocytes in the Purkinje cell layer(P)& granule cell layer(G). X 400.

In the cerebellum, the control group showed a weak positive immunohistochemical reaction for caspase-3 (Fig. 6a). The statin group exhibited strong cytoplasmic immunoreactivity (Fig. 6b), whereas the statin + THQ group showed mild cytoplasmic immunoreactivity (Fig. 6c).

**Fig. 7:**
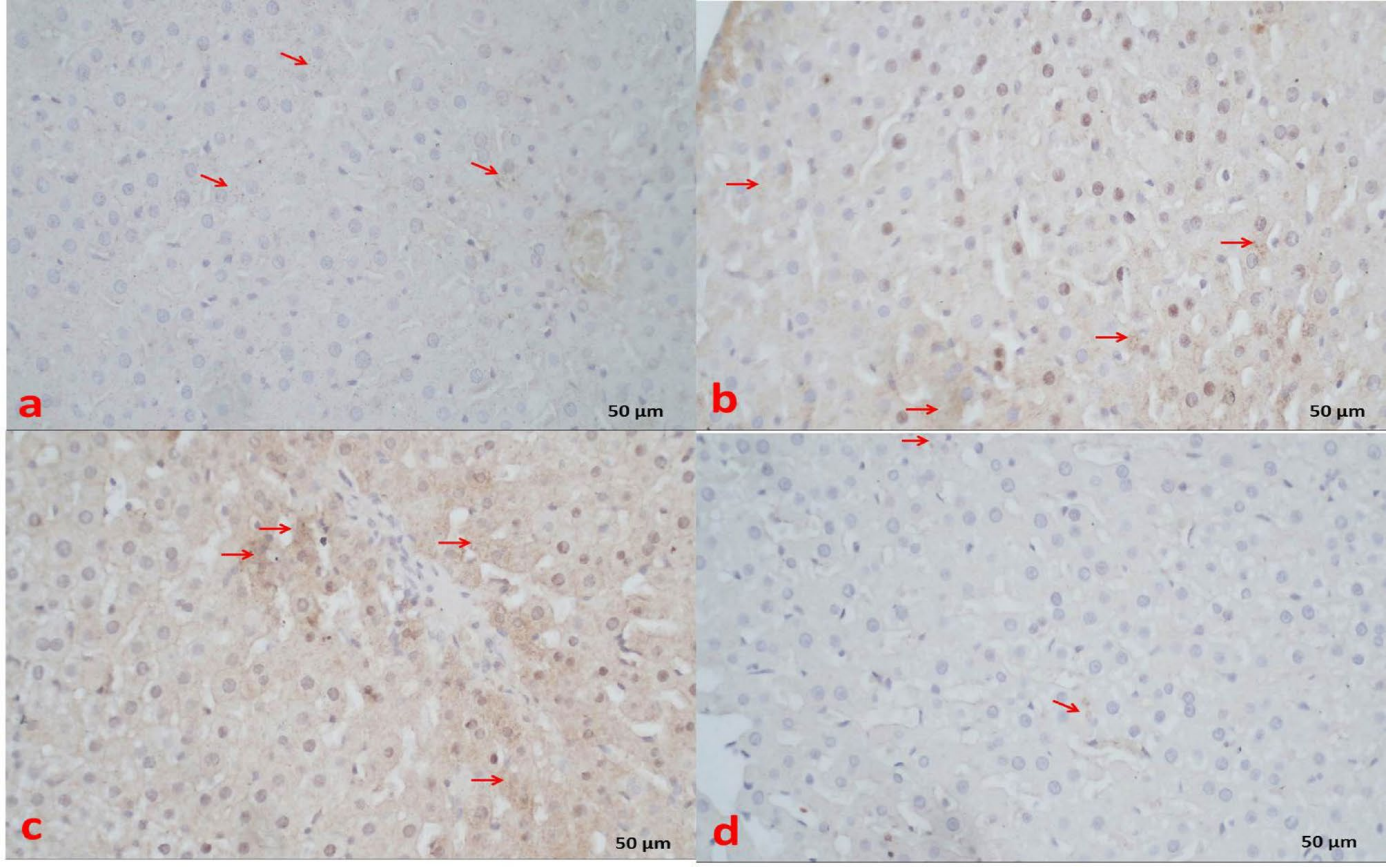
**(a)** Section of liver of control group showing hepatocytes with faint positive cytoplasmic anti caspase-3 reaction (arrows). **(b) & (c)** Section of liver of Statin group showing hepatocytes with strong positive cytoplasmic (arrows) and nuclear (n) anti caspase-3 reaction. **(d)** Section of liver of Statin + THQ group showing hepatocytes with faint positive cytoplasmic anti caspase-3 reaction (arrows) X 400.

In the liver, the control group demonstrated faint cytoplasmic anti–caspase-3 reaction in hepatocytes around the central veins (Fig. 7a). The statin group exhibited very strong cytoplasmic anti–caspase-3 reaction in hepatocytes around the central veins and in the portal area (Fig. 7b and c). The statin + THQ group showed mild cytoplasmic immunoreactivity in hepatocytes (Fig. 7d).

### Electron microscopic examination of the cerebellum

**Fig. 8:**
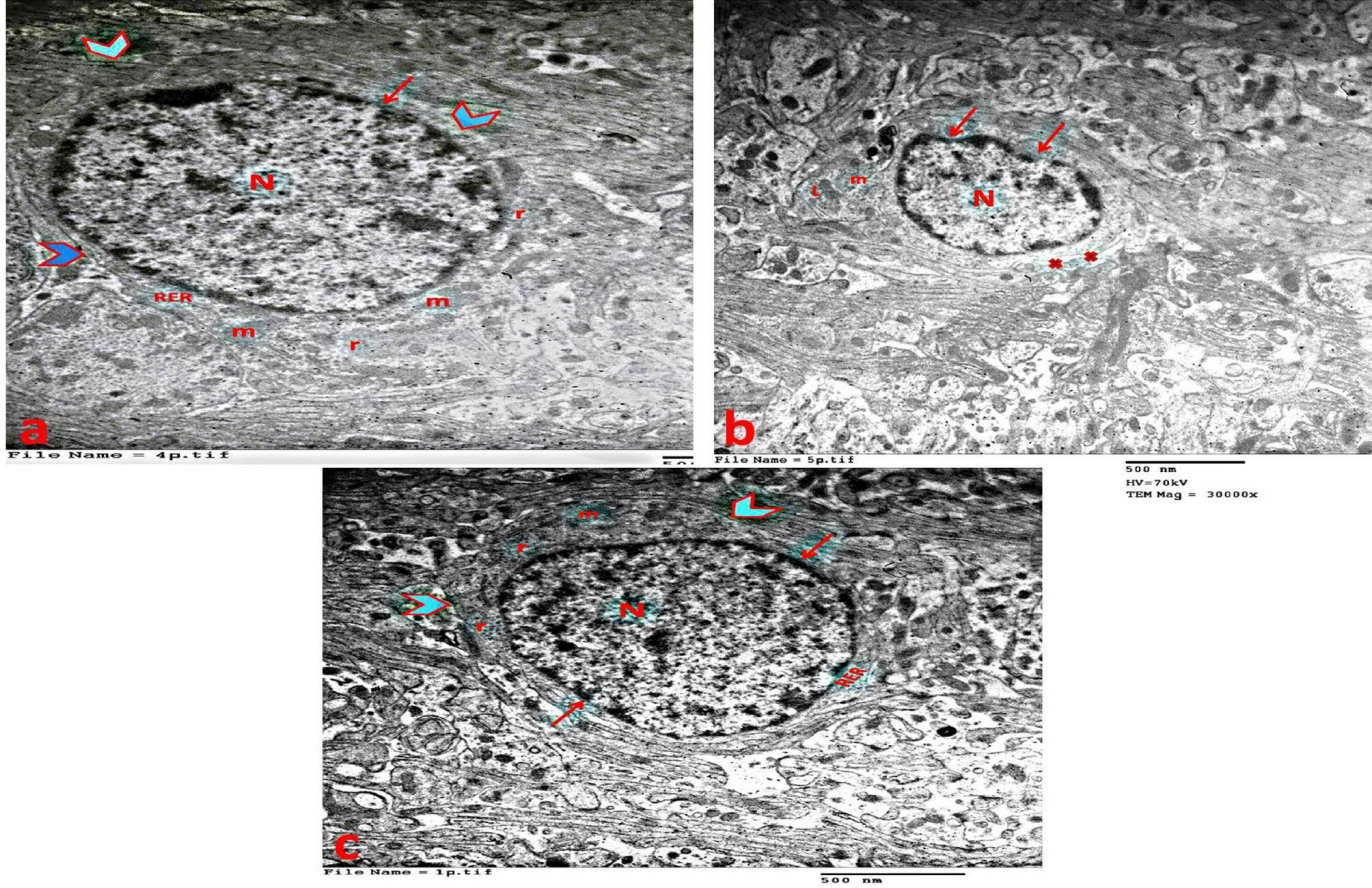
**(a)** Purkіnje cell of control cerebellar cortex group (I) with well- defined cell outline (thick arrow head) displaying euchrοmatic nucleus (N) with well-defined border (arrow), mitochondria (m), multiple rough endoplasmic reticulum cisternae (RER), and dispersed ribosomes (r) are seen in cytoplasm. **(b)** Purkіnje cell of statin group appear as shrunken cell which displaying heterochrοmatic nucleus (N) with irregular border (arrow). The cytoplasm showing damaged mitochondria (m) and 2nd lysosomes (L) with perinuclear spaces appeared around the cell (**×). (c)** Purkіnje cell of Statin + THQ group appear larger with large euchrοmatic nucleus (N) with well-defined border (arrow) and well-defined cell outline (thick arrow head), cytoplasm showing mitochondria (m), multiple rough endoplasmic reticulum cisternae (RER), and dispersed ribosomes (r) (TEM X 30000).

**Fig. 9:**
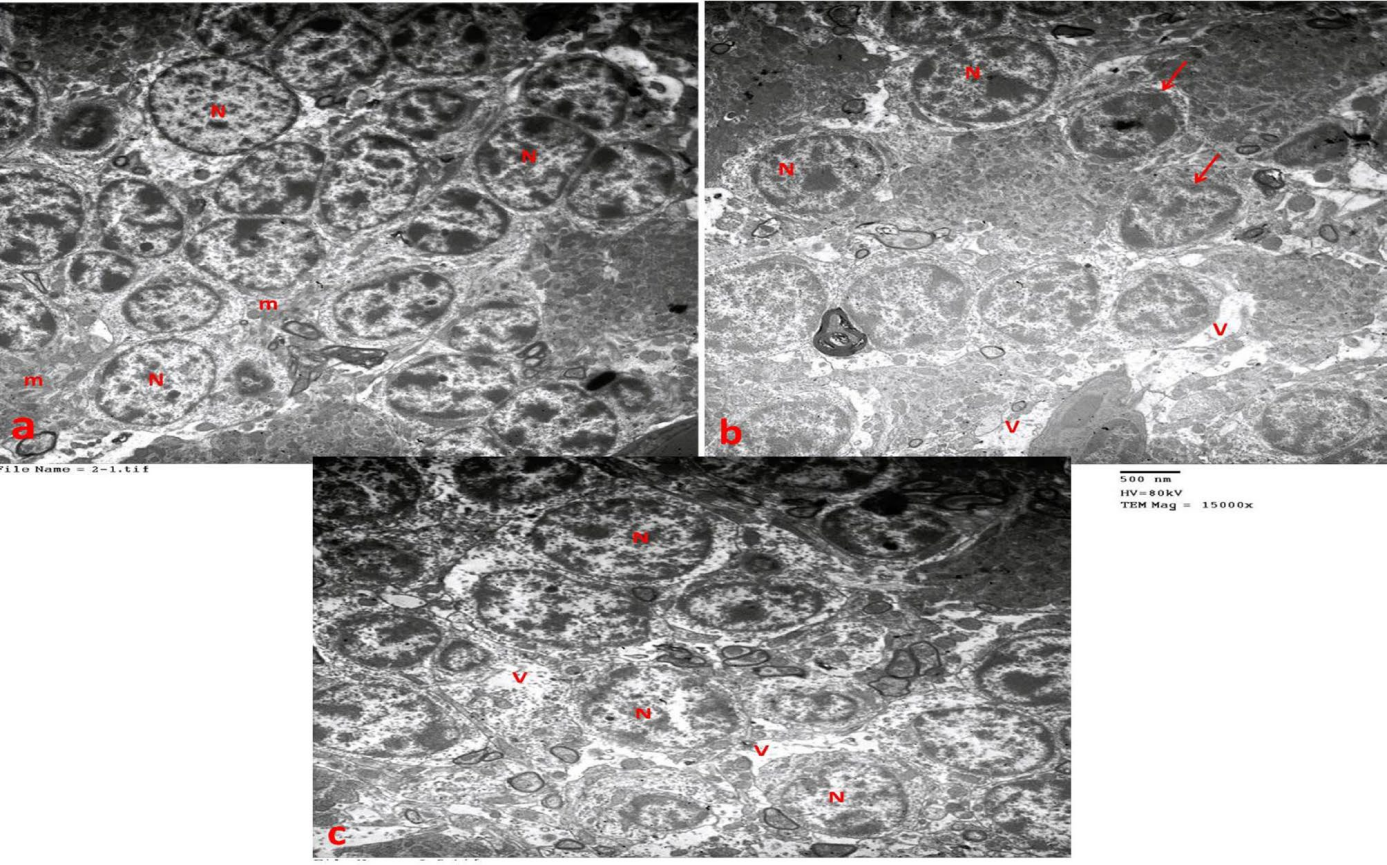
**(a)** Cerebellum of control group showing granule cells of multiple sizes having round euchromatic nuclei (N) with peripheral heterochromatin clumps encircled by a thin rim of cytoplasm having multiple mitochondria (m). **(b)** Cerebellum of Statin group showing granule cells with irregular, heterοchromatic nucleus(N) having wide perіnuclear space (arrow), multiple vacuoles appear in-between cells (V). **(c)** Cerebellum of Statin + THQ group showing granule cells with round euchromatic nucleі (N) having peripheral heterochromatin clumps, vacuoles still present in-between cells. (TEM X 15000)

In the control group, ultrathin sections revealed Purkinje cells as large flask- shaped cells with euchromatic nuclei and well-defined cell and nuclear borders. The cytoplasm contained numerous mitochondria, dispersed ribosomes, and rough endoplasmic reticulum (RER) cisternae (Fig. 8a). Densely packed granule cells were observed in the granular layer, each with a nucleus containing peripheral heterochromatin clumps surrounded by a cytoplasmic ring containing mitochondria and free ribosomes (Fig. 9a).

In the statin group, structural alterations were evident; Purkinje cells appeared shrunken with irregular, ill-defined borders, and their nuclei were irregularly heterochromatic. The cytoplasm contained dilated RER cisternae, damaged mitochondria, and secondary lysosomes (Fig. 8b). Granule cells showed irregular shapes, shrunken heterochromatic nuclei, and expanded perinuclear spaces, with multiple intercellular vacuoles (Fig. 9b).

In the statin + THQ group, Purkinje cells largely retained normal structure, with euchromatic nuclei, well-defined borders, and distinct cellular margins. The cytoplasm contained numerous RER cisternae, mitochondria, and scattered ribosomes (Fig. 8c). The granular layer consisted of closely packed cells with euchromatic nuclei, although some intercellular vacuoles persisted (Fig. 9c).

### Electron microscopic examination of the liver

**Fig. 10:**
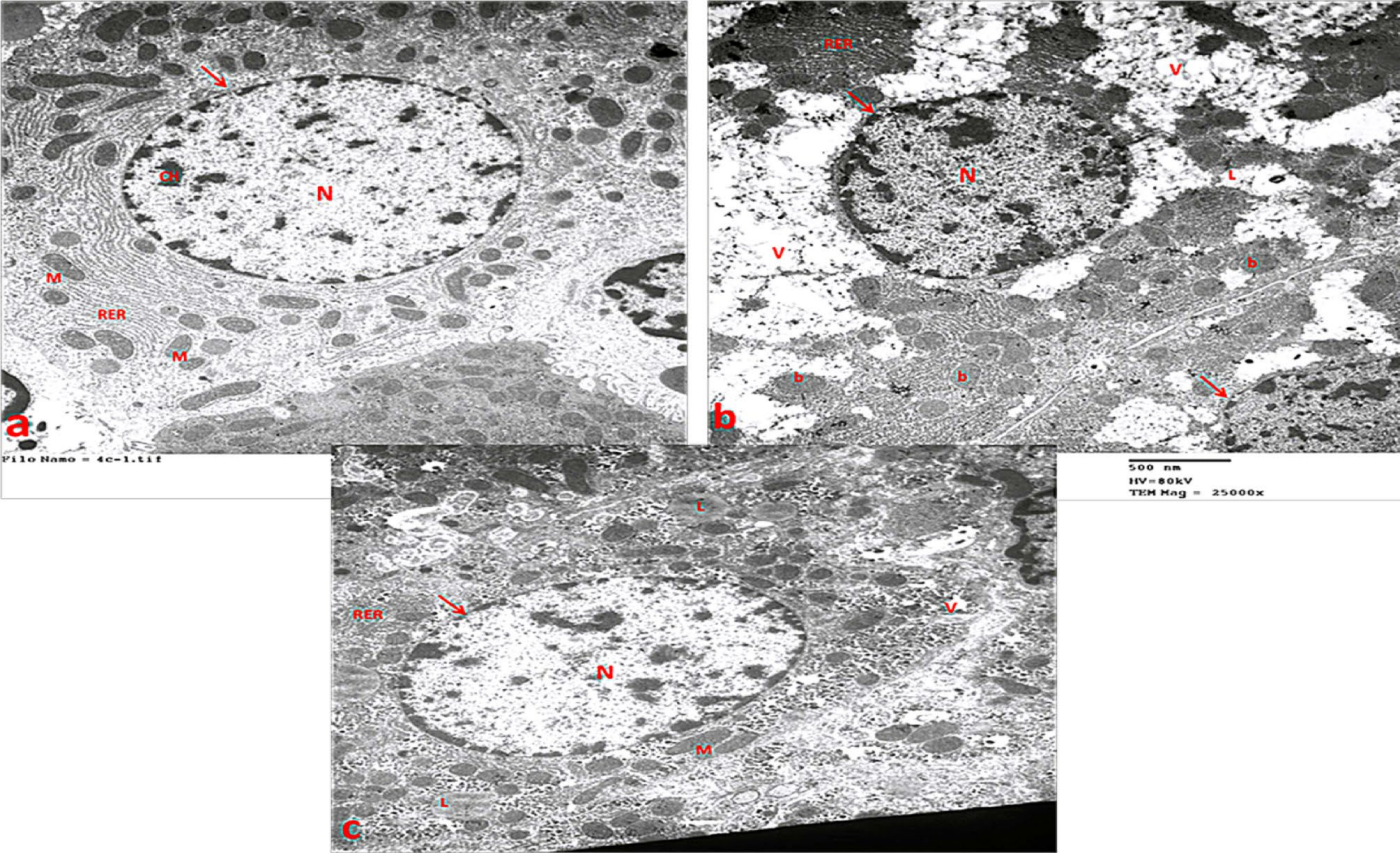
**(a)** Liver of control group showing hepatocyte, its nucleus (N) surrounded by a well-defined nuclear membrane( arrow) with chromatin masses (ch), numerous intact mitochondria (M) and a rough endoplasmic reticulum (RER) that is closely packed with parallel flattened cisternae studded with ribosomes. **(b)** Liver of Statin group showing dark pyknotic nucleus (N) of the hepatocyte cell with irregular border (arrow), ballooning (b) mitochondria, fragmented rough endoplasmic reticulum (RER), vacuoles in the cytoplasm (V) and lipid droplets (L). **(c)** Liver of Statin + THQ group showing hepatocyte nucleus (N) with a well-defined nuclear membrane (arrow), numerous intact mitochondria (M) and rough endoplasmic reticulum (RER), little vacuoles in the cytoplasm (V) and lipid droplets (L) still present. (TEM X 25000)

In the control group, hepatocytes appeared large and well-defined. The nucleus was rounded with a distinct nuclear membrane and chromatin masses. Cytoplasmic organelles were normal, with dispersed round or oval mitochondria exhibiting well-developed cristae. The RER was densely packed with parallel, flattened, ribosome-studded cisternae (Fig. 10a).

In the statin group, hepatocytes displayed irregular, pyknotic nuclei, mitochondrial swelling, numerous cytoplasmic vacuoles, fragmented RER, and scattered lipid droplets (Fig. 10b).

In the statin + THQ group, hepatocytes showed partial improvement, with large nuclei and well-defined borders. Numerous dispersed mitochondria were present, and intact RER cisternae were observed. However, cytoplasmic vacuoles and lipid droplets persisted (Fig. 10c).

### Morphometric analysis results

**Fig. 11:**
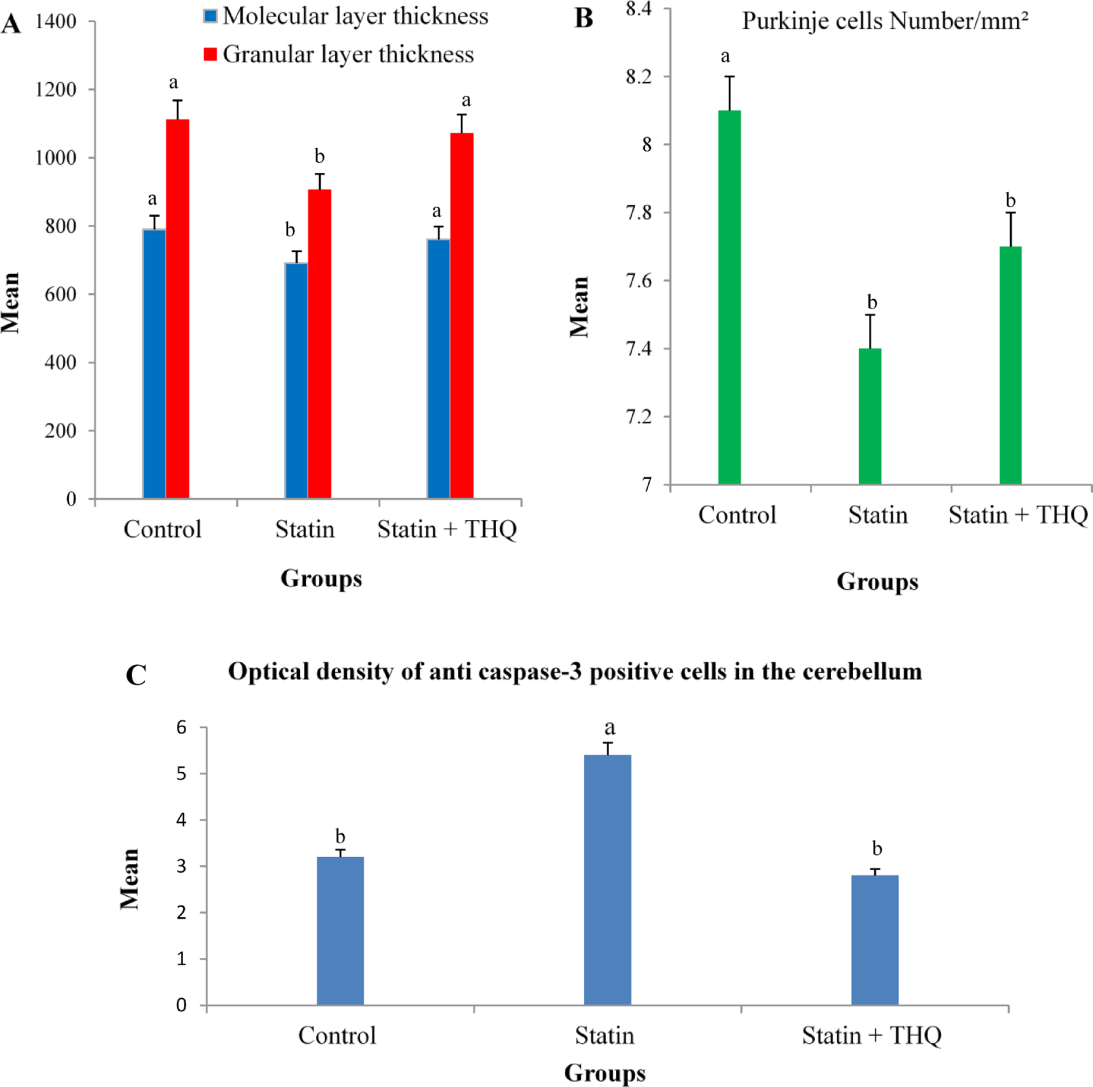
**(A)** Molecular layer thickness (MLT) & Granular layer thickness (GLT) of the cerebellum. Values with different superscripts (a & b) within the same colored column are statistically significant (*P* < 0.05). **(B)** Purkіnje cells number/mm² (PCN). **(C)** Optical density of anti caspase-3 positive cells in the cerebellum of the three experimental groups. Values with different superscripts (a & b) are statistically significant (*P* < 0.05). In all figures, values are expressed as mean ± standard deviation.

Table 1 shows morphometric changes in the cerebellum across the three tested groups. Analysis of the molecular layer thickness (MLT) revealed a significant decrease (*P* < 0.05) in the statin group compared to the Ctrl group. In contrast, the statin + THQ group showed a non-significant difference (*P* > 0.05) compared to the Ctrl group, however, it showed a significant increase (*P* < 0.05) compared to the the statin group (Fig. 11a). Analysis of the granular layer thickness (GLT) similarly revealed a significant decrease (*P* < 0.05) in the statin group compared to the Ctrl group, while the statin + THQ group showed a non-significant difference (*P* > 0.05) relative to the Ctrl group. Contrary, there was a significant increase (*P* < 0.05) in the statin + THQ compared to the the statin group (Fig. 11a).

**Table 1:** Morphometric changes of the cerebellum among the three experimental groups.

Parameters	Control group	Statin group	Statin + THQ group
Molecular layer thickness (MLT)	790.4 ± 24 ^a^	691.4 ± 49 ^b^	760.5 ± 55 ^a^
Granular layer thickness (GLT)	1112.2 ± 78 ^a^	907 ± 62 ^b^	1072.5 ± 122 ^a^
Purkіnje cells number /mm² (PCN)	8.1 ± 0.6 ^a^	7.4 ± 1 ^b^	7.7 ± 1 ^b^
Optical density of anti caspase-3 positive cells	3.2 ± 0.5 ^b^	5.4 ± 0.5 ^a^	2.8 ± 0.8 ^b^

Values are expressed as mean ± standard deviation. Values have different superscripts (a & b) in the same row are statistically significant (*P* < 0.05).

Analysis of Purkinje cell number/mm^2^ (PCN) revealed a significant decrease (*P* < 0.05) in the statin group compared to the Ctrl group. In contrast, the statin + THQ group showed a non-significant difference (*P* > 0.05) compared to the Ctrl group (Fig. 11b). Besides, there was a decrease in PCN in the statin + THQ group compared to the the statin group but it was not statistically significant (*P* > 0.05). Analysis of mean optical density of anti-caspase-3 positive cells (MODC) in the cerebellar cortex revealed a significant increase (*P* < 0.05) in the statin group compared to the Ctrl group. In comparison, the statin + THQ group showed a non-significant difference (*P* > 0.05) relative to the Ctrl group, however, it showed a significant decrease (*P* < 0.05) compared to the the statin group (Fig. 11c).

**Fig. 12:**
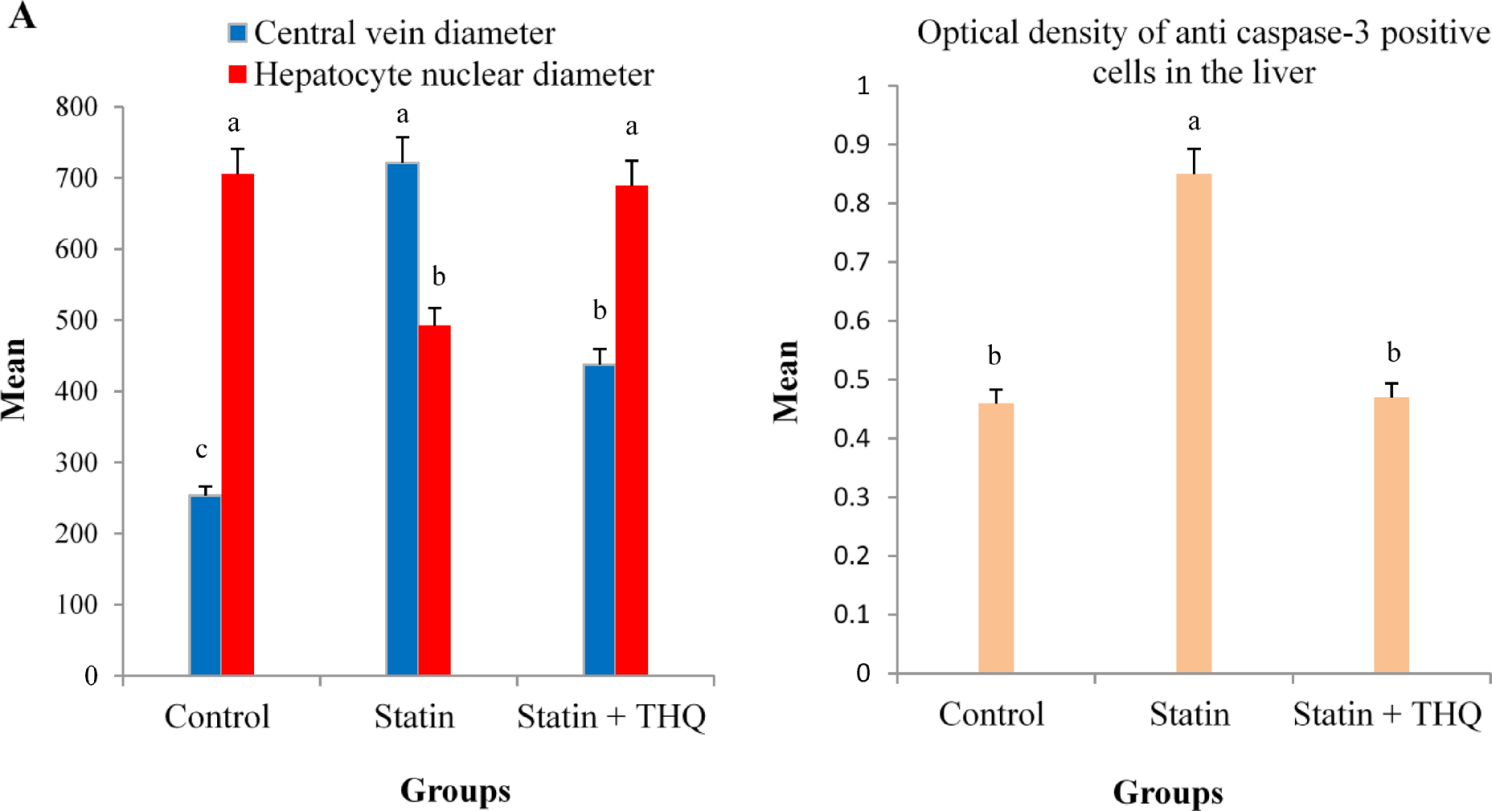
**(A)** Central vein diameter (CVD) & hepatocyte nuclear diameter (HND). Values with different superscripts (a, b & c) within the same colored column are statistically significant (*P* < 0.05). **(B)** Optical density of anti caspase-3 positive cells in the liver of the three experimental groups. Values with different superscripts (a & b) are statistically significant (*P* < 0.05). In all figures, values are expressed as mean ± standard deviation.

Table 2 shows the morphometric changes in the liver among the three tested groups. Analysis of central vein diameter (CVD) revealed a significant increase (*P* < 0.05) in the statin group compared to the Ctrl group. At the same time, the statin + THQ group showed a significant increase (*P* < 0.05) relative to the Ctrl group. Moreover, it showed a significant decrease (*P* < 0.05) relative to the statin group (Fig. 12a). Analysis of hepatocyte nuclear diameter (HND) revealed a significant decrease (*P* < 0.05) in the statin group compared to the Ctrl group. In contrast, the statin + THQ group showed a non-significant difference (*P* > 0.05) relative to the Ctrl group, but it showed a significant increase (*P* < 0.05) compared to the statin group (Fig. 12a). Analysis of the mean optical density of anti-caspase-3 positive hepatocytes revealed a significant increase (*P* < 0.05) in the statin group compared to the Ctrl group. In contrast, the statin + THQ group showed a non-significant difference (*P* > 0.05) relative to the Ctrl group, however, there was a significant decrease in statin + THQ group (*P* < 0.05) compared to the the statin group (Fig. 12b).

**Table 2:** Morphometric changes of the liver among the three experimental groups.

Parameters	Control group	Statin group	Statin + THQ group
Central vein diameter (CVD)	253.7 ± 9 ^c^	721.3 ± 28 ^a^	437.5 ± 4 ^b^
Hepatocyte nuclear diameter (HND)	705.6 ± 7 ^a^	492.4 ± 12 ^b^	689.4 ± 11 ^a^
Optical density of anti caspase-3 positive cells	0.46 ± 0.1 ^b^	0.85 ± 05 ^a^	0.47±0.02 ^b^

Values are expressed as mean ± standard deviation. Values have different superscripts (a, b & c) in the same row are statistically significant (*P* < 0.05).

## Discussion

The cerebellum is one of the vital organs of special concern in toxicological research^27^. In this study, simvastatin (20 mg/kg/day) was used, as the recommended dosage range of simvastatin tablets is 20–40 mg once daily^28^. In the present work, treatment of adult male albino rats with simvastatin for 8 weeks led to apparent changes in the cerebellum, including shrinkage of the cerebellar cortex, deeply stained Purkinje cells, an apparent increase in perineural spaces, darkly stained granule cells, and numerous vacuoles between the cells. In addition, a marked decrease in the thickness of the molecular and granular layers was observed, with strong positive caspase-3 expression in cerebellar cortical sections.

Simvastatin-induced histological alterations may have a complex origin. Previous research has shown that the monocarboxylate transporter (MCT4) preferentially absorbs statins, leading to mitochondrial dysfunction and alterations in Ca^2+^ homeostasis^29,30^. Statins (HMG-CoA reductase inhibitors) may deplete the isoprenoid pool, leading to widespread inadequate protein modification that causes organ injury. This mechanism could account for statin-induced cerebellar degenerative changes^31^.

Reduced mevalonate production, a precursor of cholesterol, leads to a decrease in cholesterol content of the sarcolemmal and mitochondrial membranes, thereby impairing membrane function and causing abnormal calcium influx^32^. Furthermore, caspase-3 is activated by elevated cytosolic calcium, which ultimately leads to cell death^33,34^. This may explain the findings of the current work observed by light microscopy.

Due to simvastatin’s ability to cross the blood-brain barrier^35^, prolonged oral administration in mice resulted in significant brain concentrations and alterations in gene expression^36^. Moreover, simvastatin significantly inhibited the robust remyelination that normally occurs following CNS demyelination induced by oral administration of the oligodendrocyte toxin cuprizone. It could induce cell death by preventing the formation of isoprenoids and cholesterol, as well as by promoting oligodendroglial process retraction through cholesterol depletion^37^.

The liver is one of the most important organs and is highly susceptible to damage caused by drug overdose or exposure to toxic agents, making it a major focus of numerous research studies^38–41^. In the present work, simvastatin treatment induced histological changes in the liver, including shrunken hepatocytes with vacuolated cytoplasm, widened blood sinusoids, dilated central veins, and strong positive expression of caspase-3. Similar results were reported in previous studies, showing that statin-induced liver fibrosis and persistent inflammation of the portal tract occurred^42^. In addition, simvastatin induced relative changes in protein secondary structure in soft tissues (liver, testis, and sciatic nerve) using a neural network approach applied to the well-known amide I protein band (1700 –1600 cm^− 1^) obtained by ATR-FTIR spectroscopy^43^. Moreover, high-dose simvastatin induced significant changes in lipid, protein, and nucleic acid content; it also increased mtDNA content and depleted protein levels in drug-treated liver tissues, suggesting oxidative stress-mediated hepatotoxicity^44^.

Caspase-3, a key marker of apoptosis, has been the focus of numerous studies for monitoring the risk of cell apoptosis and for indicating disease progression and toxicity development^45–47^. Several studies have shown that simvastatin can efficiently induce apoptosis^48–50^, which may explain the increased caspase-3 expression and destruction of hepatocytes. Ultrastructural analysis confirmed simvastatin caused cell shrinkage, swollen mitochondria, disrupted rough ER, and buildup of autophagic vacuoles in the cerebellum and liver. However, the mechanism of statin-induced ultrastructural cell degeneration is not fully understood. It may be explained by dose-related and pro-apoptotic effects, direct mitochondrial effects, pharmacological interactions, hereditary traits, or combinations thereof. Recently, an uncommon immune-mediated cell atrophy induced by statin use has been identified^51^.

Mitochondrial dysfunction is believed to be the main cause of statin-induced hepatotoxicity and neurotoxicity^52^. Numerous cellular disorders were associated with changes in autophagy and/or lysosomal function, which were linked to the presence of autophagic vacuoles^53–55^. Statins have been linked to elevated oxidative stress, which leads to mitochondrial dysfunction and transcriptional inactivation of mitochondrial biogenesis^56^.

Histological results of the statin + THQ group showed that THQ ameliorated the histological alterations caused by statin on the cerebellar cortex, with preservation of most cell shapes and sizes and a decrease in the expression of caspase-3 immunoreaction. Moreover, morphometric analysis confirmed the thickness of the molecular and granular cell layers, as well as the preservation of Purkinje cell number. It has been reported that THQ possesses strong antioxidant properties and scavenges free radical production^57^.

This provide evidence of the protective effects of thymoquinone that have been reported in various tissues. In mouse studies, thymoquinone (TQ) at a dose of 10 mg/kg/day has been frequently used as a well-tolerated pharmacological concentration that exhibits notable hepatoprotective, anti-inflammatory, and antioxidant properties, including the correction of DZN-induced hepatotoxicity. In preclinical models, it is commonly utilized to combat cancer, neurodegeneration, and oxidative stress^58^.

Oxidative damage and inflammation are thought to be the factors causing neuronal damage^59^. The imbalance between reactive nitrogen species, antioxidant enzymes, and reactive oxygen species (ROS) generation has been extensively studied in relation to oxidative stress, with numerous studies reporting its role in the initiation and progression of various diseases^60–62^. Treatment with THQ enhances antioxidant enzymes, lowers inflammation, and decreases neuronal damage. Thymoquinone may be a potential treatment for halting neuronal morphological alterations in the cerebellum^63^.

This study demonstrated improvements in liver histology following THQ treatment. This improvement was reflected in the normal appearance of most hepatocytes, the central vein, and blood sinusoids, along with a decrease in caspase-3 immunoreactivity. These findings are consistent with earlier research showing that THQ is a potent radical scavenger and has a significant impact on liver physiology^12^.

Thymoquinone has been shown to ameliorate liver damage in a variety of scenarios, including cholestatic liver disorders^64^, carbon tetrachloride hepatotoxicity^65^ and nonalcoholic steatohepatitis (NAFLD)^66^, via increasing the activity of liver enzymes, modulating the inflammatory response, and correcting oxidative status^67^. Moreover, THQ may exert hepatoprotective effects by reducing fibrogenic events, altering levels of various eicosanoids, and inhibiting reactive oxygen species generation and NF-κB signaling^68^. However, several mechanisms have been proposed to explain the antineuroinflammatory and anti-apoptotic effects of THQ. It was suggested that THQ produces anti-neuroinflammatory activity through the AMP-activated protein kinase (AMPK) and nicotinamide adenine dinucleotide (oxidized)/sirtuin 1 (SIRT1) pathways^18^. Furthermore, THQ can reduce cellular ROS generation, possibly by inhibiting the p40phox and gp91phox proteins^69^. It was stated that administration of THQ reduced infiltration of inflammatory cells, fibrosis, and collagen content due to improvements in antioxidant defenses and a reduction in oxidative damage^70^.

These findings are consistent with earlier research showing that THQ is a potent radical scavenger and has a significant impact on liver physiology^12^. THQ has been shown to ameliorate liver damage in a variety of scenarios, including cholestatic liver disorders^64^, carbon tetrachloride hepatotoxicity^65^, and nonalcoholic steatohepatitis (NAFLD)^66^, via increasing the activity of liver enzymes, modulating the inflammatory response, and correcting oxidative status^67^. Moreover, THQ may exert hepatoprotective effects by reducing fibrogenic events, altering levels of various eicosanoids, and inhibiting the generation of reactive oxygen species and NF-κB signaling^68^.

Ultrastructural results of the statin + THQ group in the current trial in both the cerebellum and liver confirmed histological improvement, with restoration of cell shape, size, and cytoplasmic organelles. In a prior study, rats treated with tramadol+*Nigella sativa* showed fewer ultrastructural apoptotic alterations in the cerebral cortex than those treated with tramadol alone. Most neurons and axons appeared normal, with intact mitochondria and rough endoplasmic reticulum^71^.

Thymoquinone was reported to reverse brain damage, particularly that induced by mineral toxicity such as sodium nitrate, through several mechanisms, including lowering oxidative stress, restoring glutathione levels, blocking pro-inflammatory cytokines, restoring cytochrome c-oxidase activity, and reducing apoptosis markers in rat brain tissues^72^.

The antioxidant properties of thymoquinone were the primary driver of its neuroprotective effects; therefore, it is a valuable tool for mitigating the adverse effects of both acute and chronic forms of brain disease. This improvement occurs by restoring normal levels of antioxidant enzymes and halting the increase in lipid peroxidation products. These antioxidant properties make this substance a promising basis for the development of prototype therapeutic agents aimed at the treatment of several degenerative diseases of the central nervous system^73^.

The liver improvement results of the present study are consistent with previous research^74^, which stated that rats given THQ showed fewer histological and ultrastructural alterations in the liver and testes than the treated group. Bouhlel et al. (2017) showed that THQ protected rat liver against ischemia-reperfusion injury by preventing endoplasmic reticulum (ER) stress and mitochondrial dysfunction^75^. These effects implicate the prevention of oxidative stress.

Furthermore, these findings are consistent with previous research^74^, which stated that rats given THQ showed fewer histological and ultrastructural alterations in the liver and testes than the treated group. Thymoquinone is a strong antioxidant and free radical scavenger, which explains these effects.

Thymoquinone supplementation was found to significantly protect multiple organs against oxidative damage induced by various free radical-generating agents, including aflatoxin B1-evoked hepatotoxicity, gentamicin-induced nephropathy, and ethanol-induced gastric mucosal injury^76–78^. Its high potency and low systemic toxicity render THQ an encouraging alternative to conventional therapeutic drugs^79^.

In this study, thymoquinone was administered at a dose of 10 mg/kg/day for 8 weeks, a dosage that was both safe and effective. This regimen demonstrated notable protective effects on both the cerebellum and liver, organs in which such effects are typically observed only after prolonged treatment. Thymoquinone acted as a potent anti-inflammatory and antioxidant agent, thereby preserving cerebellar structure and cognitive function while improving liver function. These findings highlight the novel aspects of the present study, which were substantiated through combined ultrastructural and morphometric analyses of the cerebellum and liver.

The limitations of the study include the absence of a group receiving thymoquinone alone and the lack of direct measurements of biochemical markers of oxidative stress, such as malondialdehyde (MDA), superoxide dismutase (SOD), catalase (CAT), or glutathione (GSH). In addition, the assessment of apoptosis was limited to caspase-3 expression, which does not provide a comprehensive evaluation of apoptotic pathways.

## Conclusion

Simvastatin induced significant degeneration in both the cerebellar cortex and liver tissue, primarily due to increased oxidative stress and reduced antioxidant protection. Thymoquinone exerted protective effects against oxidative damage in both organs and alleviated degenerative changes induced by statins.

## Data Availability

All data generated or analyzed during this study are included in this published article.
